# Exploring the Roles of Body Dissatisfaction, Cognitive Distraction, and Age in Sexual Distress Related to Sexual Function and Sexual Satisfaction in Men: An Extended Understanding Using a Moderated Mediation Model

**DOI:** 10.3390/healthcare13070843

**Published:** 2025-04-07

**Authors:** Ivanilda B. Costa, Pedro J. Rosa, Patrícia M. Pascoal

**Affiliations:** 1 HEI-Lab—Digital Human-Environment Interaction Labs, Lusófona University, Campo Grande 376, 1749-024 Lisboa, Portugal; ivanilda.costa@ulusofona.pt (I.B.C.); pedro.rosa@ulusofona.pt (P.J.R.); 2 Instituto Superior Manuel Teixeira Gomes (ISMAT), Rua Dr. Estevão de Vasconcelos 33a, 8500-590 Portimão, Portugal; 3 Clínica Universitária de Psiquiatria e Psicologia Médica, Faculdade de Medicina, Universidade de Lisboa, Avenida Professor Egas Moniz, 1649-028 Lisboa, Portugal; 4 PSYLAB, Instituto de Saúde Ambiental (ISAMB), Faculdade de Medicina, Universidade de Lisboa, Avenida Professor Egas Moniz, 1649-028 Lisboa, Portugal

**Keywords:** body dissatisfaction, cognitive distraction during sexual activity, sexual satisfaction, sexual distress, path analysis, moderated mediation

## Abstract

**Background/Objectives**: Self-objectification theory posits that objectification of people’s bodies, as a synonym for self-worth, translates into body surveillance and dissatisfaction, which has a negative impact on both social and emotional adjustment. According to empirical data based on cognitive models, body dissatisfaction translates into cognitive distraction during sexual activity, affecting sexual response. However, the association of body dissatisfaction with other sexual outcomes, such as satisfaction and distress, in heterosexual men is understudied in comparison to women. **Methods**: This observational, cross-sectional, and correlational study used a convenience sample of 597 heterosexual males with a mean age of 35.52 (SD = 8.78) obtained through a web survey. **Results**: Results suggested that cognitive distraction mediated the association between body dissatisfaction, sexual distress, and satisfaction. A moderating effect of age was found, detecting a decrease in the mediated effect of cognitive distraction as men aged, reinforcing the idea of age as a protective factor. **Conclusions**: Our study further supports cognitive models that are useful to understand sexual outcomes and not merely sexual function and reinforce the existence of heterosexual men’s body dissatisfaction and its detrimental effects, suggesting that health practitioners should assess this phenomenon in a context related to sexual health.

## 1. Introduction

### 1.1. Body Image

Body image was initially conceptualised as one-dimensional and defined as how a person perceives their body [1]. However, it has been gradually defined as a multidimensional construct that encompasses affective (i.e., shame and dysphoria), behavioural (i.e., avoidance and concealment) and cognitive (e.g., dissatisfaction and desire for change) dimensions that everyone feels regarding their perceived physical appearance [1,2]. According to Cash et al. [3], body image refers to one’s perceptions and attitudes about their body, especially their physical appearance [3]. Cash [4] considers that body image has three dimensions: investment, which denotes the importance one places on physical appearance as well as the effort one is willing to put into achieving it, affect, referring to the emotional experiences that result from body-related evaluations, and evaluation, which refers to the feelings of satisfaction or dissatisfaction with different aspects of one’s appearance.

Most studies done in this field have been about the negative aspects of body image, focusing on body image dissatisfaction, or simply body dissatisfaction, which is associated with psychological disorders (e.g., eating disorders and depression) [5,6]. Body dissatisfaction seems to be higher among women than men across ages [7].

These associations are seen in cisgender men and women [8] as well as transgender people [9]. Even though body dissatisfaction seems to be studied in different populations, there are more studies linking it with sexual functioning (e.g., satisfaction, orgasm, and pain) with samples of women [10] and some studies suggest that body dissatisfaction and its detrimental effects are more common among women than men [11].

Recent studies suggest that body dissatisfaction can be experienced in qualitatively diverse ways among men and women [12]. For example, “fat talk” (talking about people’s body shape and fitness, including perceived fatness) in women is centred on thinness, talking negatively about different body parts. At the same time, men tend to speak more about muscularity and more positively about other body parts [13]. The desired body seems to change among men, with younger men wanting a more muscular body and older men a leaner body, while in women it seems stable and related to societal norms [7].

Self-image, whether in men or women, tends to be stable through time yet malleable to the impact of context variables [14]. Body image concerns can be intensified in situations where one’s body is in the spotlight [8,15]. Cohane and Pope Jr.’s review [16] found evidence that body image-related concerns start early in men. Because of the precocity of these concerns among men, studying this phenomenon in this population becomes pertinent, since the existing studies are scarce.

In a study with LGB+ cisgender women and men, it was found men tend to be as satisfied with their appearance and as distracted about their bodies in intimate situations as women, although body appearance cognitive distraction during sexual activity mediated the relationship between body dissatisfaction and sexual satisfaction only in the men’s sample [17]. However, we do not know if it is the same for heterosexual men as the association between body dissatisfaction and sexual satisfaction through the effect of cognitive distraction while examining the potential moderating effect of age has not been studied in this group.

In women samples, such as in Tiggemann and McCourt’s study [2], older women presented higher levels of body appreciation, a construct defined as a positive body image that is related to body satisfaction, than younger ones, and the association between body appreciation and body satisfaction/dissatisfaction diminished as age increased. Since it was verified that body appreciation differs throughout women’s lifespans, studying how body image-related dimensions will be experienced amongst men in different age frames becomes more imperative. This will allow us to understand better which variables could be influencing the phenomenon in this population, namely if age influences how body image-related concerns are expressed, as well as the effects these concerns could have on how men experience sexual activity (with or without satisfaction).

The negative experience of body image/body dissatisfaction (e.g., body shame and weight distortions) has been associated with an increase in risky sexual behaviours, lower sexual self-esteem, lower frequency of sexual behaviour, lower sexual desire, and sexual dysfunction, whether in men or women [10,11]. Despite the consensus about the significant association between body dissatisfaction and some dimensions of human sexuality, little is known about the association between body dissatisfaction and two of the main outcome variables on sexual health: sexual satisfaction and distress.

### 1.2. Sexual Satisfaction

According to the World Health Organization (WHO), sexuality is a central aspect of being human that is experienced and expressed in terms of thoughts, fantasies, desires, beliefs, attitudes, values, behaviours, practices, roles, and relationships [18]. Even though sexuality can include all the dimensions previously referred to, not all of them will be experienced or expressed by every person and sexuality is influenced by the interaction of economic, political, cultural, ethical, legal, historical, religious, biological, psychological, social, and spiritual factors [18]. The WHO understands that sexual health is a state of physical, emotional, mental, and social well-being which requires a positive and respectful approach to sexuality and sexual relationships and the possibility of having pleasurable and safe sexual experiences, free of discrimination, coercion, and violence [18], and sexual satisfaction is one of the sexual health indicators [19]. The concept of sexual satisfaction has different definitions, with Lawrance and Byers’s [20] being the most widely used, defining it as an affective response that emerges from the subjective evaluation one makes of the negative and positive dimensions of one’s sexual relationship, that is, the measure in which a person is satisfied with their sex life [20]. It is considered an important component of human sexuality, being seen as the goal of the sexual response and a sexual right [21]. It is considered a key factor in one’s quality of life since it is associated with a better state of psychological and physical health, overall well-being, and quality of life [19,21,22]. It is known that sexual satisfaction decreases with age [23], but the different factors that may modulate this decrease and their interaction need to be better explored.

Stephenson and Meston [19] evaluated sexual satisfaction and sexual distress in a sample of women and found that even though these two outcomes of sexual health were associated with sexual functioning, sexual distress was more closely related to sexual functioning in the clinical sample, and this association decreased with clinical intervention. As this study highlights, the sexual distress and satisfaction constructs are significantly statistically associated in both the clinical and the nonclinical samples. The authors concluded, based on their results, that although partially associated, the constructs are independent [19]. In conclusion, sexual satisfaction presents an important relationship with a clinically significant indicator—sexual distress [19].

### 1.3. Sexual Satisfaction and Body Image

As previously stated, studies have been suggesting an association between the evaluation of one’s body image and sexual satisfaction, with an association found between poor body image and different aspects of sexuality, e.g., sexual avoidance, difficulty in reaching orgasm, low sexual satisfaction, an association that seems to persist despite the effects of one’s actual weight [8,11,24].

Furthermore, studies suggest that negative body image can negatively affect sexual functioning by increasing self-consciousness during sexual activity, which is related to cognitive absorption with concerns related to how a sexual partner perceives one’s body during physical intimacy, which can decrease attention to positive internal states, e.g., sexual arousal and physical pleasure, influencing negatively one’s sexual functioning [9]. In line with a study by Meana and Nunnink [25] that found that in both men and women, body image could be a strong predictor of body image cognitive distraction, Daniel and Bridges’ study with men [15] found that none of the variables of body image (e.g., body shame, drive for muscularity, and body surveillance) had that role by itself in their sample. However, masculinity (evaluated through gender roles) was the only variable identified as a statistically significant predictor of sexual satisfaction.

Milhausen et al. [8] found that the three dimensions of body image (behavioural, affective, and specific to the sexual encounter) among men were associated with sexual satisfaction even after the effect of relationship satisfaction was controlled. Body image evaluation during a sexual encounter, a form of cognitive distraction, was the only specific dimension that influenced sexual functioning. Men with more significant body fat were likelier to have a negative body image in the behavioural and affective dimensions [8].

None of the studies mentioned above considered age as valuable information, which could be important considering that self-objectification in men has been increasing in the last several decades [12]. If, on the one hand, we could expect younger men to be more vulnerable to concerns about body image, on the other hand, their image is closer to the social standards of what a beautiful male body is. In this line, it can be helpful to better understand the interaction between male body image and sexual outcomes (both positive and negative) in different age frames.

### 1.4. Sexual Distress

The existence of sexual difficulties is a common experience among the general population; however, when sexual problems are persistent, they tend to be associated with lower self-esteem and feelings of well-being, marital distress, and lower levels of happiness [26]. Marked sexual distress, which involves worry, anxiety, and frustration regarding one’s sexual activity, is necessary for a diagnosis of sexual dysfunction, according to the Diagnostic and Statistical Manual of Mental Disorders [19]. Therefore, it is important to understand which factors affect sexual distress related to sexual function as a clinically meaningful experience [27].

#### 1.4.1. Sexual Distress and Cognitive Distraction During Sexual Activity

When referring to the concept of cognitive distraction during sexual activity (from now on, it will be referred as cognitive distraction), Masters and Johnson’s 1970 [28] construct of *spectatoring* must be mentioned, a construct that was later developed in Barlow’s 1986 [29] cognitive–affective model of sexual dysfunction.

This concept implies that by observing and monitoring one’s actions during sexual activity, one distracts oneself from sexual sensations and cues, which can compromise one’s sexual functioning [26,30]. Masters and Johnson [28] affirmed that, by focusing on the quality of their erectile function during sexual activity, men would be more prone to have performance-related cognitive distraction, and Barlow’s 1986 [29] cognitive–affective model of sexual dysfunction for men suggests that the difference between functional men and dysfunctional men rests on these functioning-related thoughts, failure, and the consequences of it. A recent review on the role of cognitive processing factors in men’s and women’s sexual function has demonstrated the pivotal role that cognitive distraction has on both men’s and women’s sexual function [31].

Studies in this field suggest cognitive distraction’s contents are different between men and women, with men presenting more performance-related cognitive distraction and women more appearance-related cognitive distraction [26]. In Silva et al.’s study [32], the authors found that there were no gender differences between levels of beliefs about appearance and cognitive distraction shown by participants and confirmed that cognitive distraction was negatively associated with sexual functioning and that it mediated the association between beliefs about appearance and sexual functioning. These results suggest that the more a person, self-identified as cis man or cis woman, believes that their appearance determines their personal and interpersonal success, the more distracted a person will become with their body during sexual activity [32]. This study sustains that body image is important for men’s sexual function, but one of its limits is the absence of dimensions of people’s sexual dysfunction: sexual distress.

Previous research has shown that body dissatisfaction mediated by body image cognitive distraction affects cis women’s experience of sexual distress related to sexual function [33]. The study by Carvalheira et al. [34] about the impact of body image dissatisfaction on sexual functioning distress corroborated the detrimental effects of appearance-related cognitive distraction on sexual distress and observed that among men, anxiety during sexual activity caused them great distress when compared to women. A developmental approach that looks at differences across age frames is necessary to understand how these associations may evolve.

#### 1.4.2. Age and Sexual Distress

Due to the ageing process and sexual distress, studies with adults over 65 years old indicate that sexual dysfunction and problems are common in this age frame [35]. They are affected by both psychological and interpersonal factors. However, even though a lot of these adults experience sexual difficulties, these difficulties do not cause them distress [36]. From a clinical standpoint, it is relevant to understand better which factors affect sexual distress in different ages. As there is an imbalance in the research developed with men and women, with most research being developed with samples of women, we aim to give our contribution on an understudied topic in the literature: the role of body image dissatisfaction in men’s distressful sexual function in different age frames.

### 1.5. Research Questions and Hypotheses

Considering the existing literature concerning the association between body image, cognitive distraction during sexual activity, sexual satisfaction, and distress, we propose the following research hypotheses (RH):

**RH 1.**  *In this study, the measures of central tendency for body image, cognitive distraction during sexual activity, sexual distress, and sexual satisfaction will have similar values as the ones found in similar studies.*

**RH 2.**  *The study variables (body dissatisfaction, cognitive distraction, sexual distress, and sexual satisfaction) will have significant associations between them.*

**RH 3.**  *Cognitive distraction mediates the association between body satisfaction, sexual satisfaction, and sexual distress.*

Since we did not find previous empirical studies that would allow us to support a hypothesis, we will place the following exploratory research question (RQ): Will the variable of age be a moderator for the associations found?

## 2. Materials and Methods

### 2.1. Participants

The sample for the present study was drawn from a larger project involving 779 self-identified heterosexual Portuguese adult men aged 18 years or older, regardless of their current romantic relationship status. The project was approved by an ethics committee for research in our university—the Ethical and Deontological Committee for Scientific Research at Lusófona University—and participants did not receive an incentive to complete the survey. The eligibility criteria established that to be included in this study, people needed to identify as a cis woman or cis man (a definition was provided and an explanation regarding the cis-centred nature of the measures used), be fluent in Portuguese, be at least 18 years old, and be engaged in an exclusive, committed romantic dyadic relationship, as this was a prerequisite for responding to questions related to sexual satisfaction and not have missing data on items assessing sexual satisfaction and distress. A total of 182 participants were excluded for failing to meet the eligibility criteria, with 154 excluded based on the first criterion and 32 based on the second. The final sample, comprising 597 participants aged 20 to 65 years (M = 35.52, SD = 8.78), serves as the focus of the present study. According to Fritz and MacKinnon [37], our final sample size is adequate for detecting small mediation effects, as they recommend at least 462 participants. Additionally, Preacher and Hayes [38] advise >500 participants for bootstrap confidence intervals, and guidelines for moderated mediation [39,40] support a minimum of 500 participants. Our sample of 597 exceeds these recommendations, ensuring sufficient power for our analysis. Participants ’sociodemographic data are described in Table 1.

### 2.2. Procedure

An online survey was originally developed on a dedicated website to support the study but the content was later imported to Qualtrics (https://www.qualtrics.com) (due to its robust data management capabilities and seamless integration with IBM-SPSS, which minimised transcription errors and ensured respondents could not alter the survey [41]). To ensure the survey’s quality and guarantee the process’s reliability and validity, we utilised the Qualtrics editor to evaluate the survey design. The survey received a “great” rating on the Expert Review feature [42]. Before launching the study in November 2010, a pilot test was conducted to refine the survey’s instructions and language and identify and address minor grammatical errors and typographical issues, culminating in the final version.

The study utilised a non-probabilistic snowball sampling method due to the practical constraints of web-based surveys. This sampling method relies on voluntary participation, making randomisation challenging [43]. However, it offers efficient data collection, broader geographic reach, and increased sample diversity [44]. Additionally, web-based surveys ensure anonymity, reducing social desirability bias and enabling more honest responses, particularly on sensitive topics [45]. The web-based survey was disseminated through various social media platforms to recruit participants. Potential respondents were encouraged to complete the survey and share it further, forming a non-probabilistic sample. Before participation, individuals were invited to review and provide informed consent. Data access was limited to the research team and identifying details such as IP addresses and geolocation information were removed. The informed consent form explicitly stated that the collected data would remain confidential, not be shared, and be deleted 15 years after data collection. Only men were selected for the present study because of the reduced number of studies about men in this field when compared to the vast literature about this theme in women, e.g., [24,46], and the need to create a concise, relevant study.

### 2.3. Data Preparation and Statistical Analysis

As participants were required to complete each question before submission, we had no missing data. Firstly, the participants’ responses to the instruments employed in the study were characterised using measures of central tendency (mean) and dispersion (standard deviation, minimum, and maximum).

Secondly, in order to identify potential confounding variables, we regressed body satisfaction (predictor), cognitive distraction (mediator), and both sexual distress and sexual satisfaction (both outcomes) on relationship status, education, ethnicity, and socioeconomic status. As no statistically significant results were found (all *p*_s_ > 0.05), none of these variables were included in our statistical model. After, an exploratory analysis was performed to evaluate the theoretical assumptions required for parametric tests, including Pearson’s bivariate correlation and linear regression. Subsequently, a reliability analysis was performed using Cronbach’s alpha, interpreted according to DeVellis’ [47] criteria.

**Figure 1:**
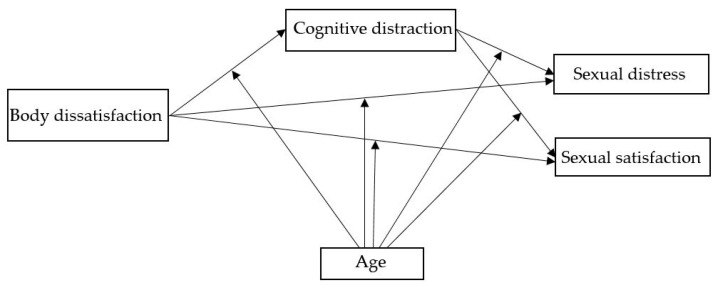
Proposed conceptual model.

Finally, the relationship among the study’s variables was assessed and an ordinary least squares (OLS) path analysis was conducted. Prior to testing the mediation and moderated mediation models, scale scores were standardised using Z-transformations to derive z-scores from the unstandardised beta coefficients. Separate models were analysed for each outcome variable—sexual distress and sexual satisfaction. The mediation effect (indirect effect) was tested with the bootstrapped 95% confidence intervals (CIs) with 10,000 resamples. The effect of X on Y was assessed at −1 SD, 0 SD, and +1 SD of the moderator (age) [48]. Mediation and moderated mediation effects were examined as depicted in Figure 1. The residual variance for each outcome variable was derived from the Sums-of-Squares and Cross-Products (SSCP) matrices, and the residual correlation between the outcome variables was subsequently computed following the guidelines outlined by Tabachnick and Fidell [49].

For the moderated mediation model, the following conditions were considered: (1) the effect of the independent variable on the mediator depends on the moderator and the effect of the mediator on the criterion variable is significant, or the effect of the mediator on the criterion variable depends on the moderator and the effect of the independent variable on the criterion variable is significant; (2) the conditional indirect effect of the independent variable on the criterion variable through the mediator depends on specific values of the moderator. The second condition is essential for demonstrating moderated mediation [50].

In terms of effect size, the completely standardised indirect effect (ab_cs_) was interpreted according to the criteria proposed by Preacher and Kelley [51]: small (0.01), medium (0.09), and large (0.25). The total mediated effect proportion (MP) was analysed based on the guidelines provided by Shrout and Bolger [52].

Descriptive and correlational analyses were conducted using IBM SPSS Statistics version 24 (SPSS Inc., Chicago, IL, USA) for Windows. The significance level was set at 5% (*p* < 0.05). The mediation and moderated mediation analyses (model 4 and model 59, respectively) were conducted using PROCESS macro version 3.3 developed by Hayes [50].

### 2.4. Measures

#### 2.4.1. Sociodemographic Data

A brief sociodemographic questionnaire was used to collect information on age, relationship status, education, ethnicity, socioeconomic status, and area of residency.

#### 2.4.2. Global Body Dissatisfaction Scale (GBDS)

The GBDS is a subscale of the Body Attitude Test; it has four items and was developed by Probst et al. [53]. The GBDS is a general measure that evaluates body image dissatisfaction based on the frequency of negative perceptions, behaviours, and feelings one feels about their own body (e.g., “When I look at myself in the mirror, I’m dissatisfied with my own body”). It is answered on a six-point Likert’s scale—ranging from 1 = “never” to 6 = “always”. Total score can range from 4 to 24 points, with higher scores meaning higher levels of body image dissatisfaction. This measure has shown high validity and reliability values in Portuguese samples (e.g., α = 0.82 in Pascoal et al.’s [54] study (Portuguese validation; α = 0.86 for the male sample). In the current study, the GBDS presents an α = 0.77, demonstrating an acceptable reliability.

#### 2.4.3. Body Appearance Cognitive Distraction Scale (BACDS)

The BACDS is a subscale of the Cognitive Distraction Scale developed by Dove and Wiederman [55] and has 10 items. The BACDS assess body image cognitive distraction during sexual activity (e.g., “It is difficult to enjoy sex because of my concerns over how appealing my body is to my partner”) [29]. Participants answer the questions on a six-point Likert scale (ranging from 1 = “never” to 6 = “always”) regarding how recurrent a specific statement will be true while involved in sexual activity (e.g., “During sexual activity, I am worried about how my body looks to my partner.”). Total scores may range from 10 to 60, where higher scores indicate higher levels of body image cognitive distraction [29]. In the original study, the BACDS had a good reliability (α = 0.95), which was also found in Portuguese studies (Cronbach’s α > 0.80) [29]. In the present study, the BACDS showed a Cronbach’s alpha (α) of 0.86, indicating good reliability.

#### 2.4.4. Sexual Distress Related to Sexual Function (NATSAL-SF)

To measure sexual distress related to sexual function, we used 7 questions extracted from the National Survey of Sexual Attitudes and Lifestyles (Natsal) questionnaire, which evaluates sexual functioning (NATSAL-SF) [56]. Participants are asked to indicate, for each potential sexual functioning difficulty, which they have experienced in three months in the past year: (1) lack of sexual interest, (2) inability to feel aroused, (3) inability to maintain the arousal, (4) premature orgasm, (5) decreased orgasm, and (6) anorgasmia. Furthermore, distress was measured by asking participants how stressful experiencing sexual difficulties was for them, and the answer was given on a six-point Likert scale (0 = “Not having any sexual difficulty”, 1 = “Not distressful”, 2 = “Somewhat distressful”, 3 = “Quite distressful”, 4 = “Extremely distressful”). The total score could range from 0 to 30 points, with higher scores indicating higher levels of sexual distress. Cronbach’s alpha was not assessed, following previous usual procedures using different versions of this measure [33,56].

#### 2.4.5. Global Measure of Sexual Satisfaction (GMSEX)

The GMSEX is a measure developed by Lawrance and Byers [20] to evaluate sexual satisfaction in men and women through subjective appreciation that one has regarding their sexual relationship with a current partner [54].

This measure has five items that characterise the relationship in a bipolar way on a seven-point Likert scale e.g., (1—“very bad” to 7—“very good”). The GMSEX has demonstrated stable and consistent psychometric properties, with a Cronbach’s α of 0.90 in the original study. In the Portuguese population, Pascoal et al. [54] reported an α = 0.83 for the non-clinical sample, α = 0.93 for the clinical sample, and α = 0.94 for the online sample, which indicates good reliability levels. We obtained an α = 0.94, suggesting the potential for scale reduction.

## 3. Results

### 3.1. Participants’ Responses to Instruments

To characterise participants’ responses to instruments employed in the present study, measures of central tendency and dispersion were analysed, revealing that, overall, participants reported satisfaction with their body image, their outcomes, and their sexuality. They presented low levels of sexual distress, as shown in Table 2.

### 3.2. Relationship Between the Study Variables

The association between body dissatisfaction, sexual satisfaction and distress, cognitive distraction, and age were examined (Table 3).

### 3.3. Mediation Model

**Figure 2:**
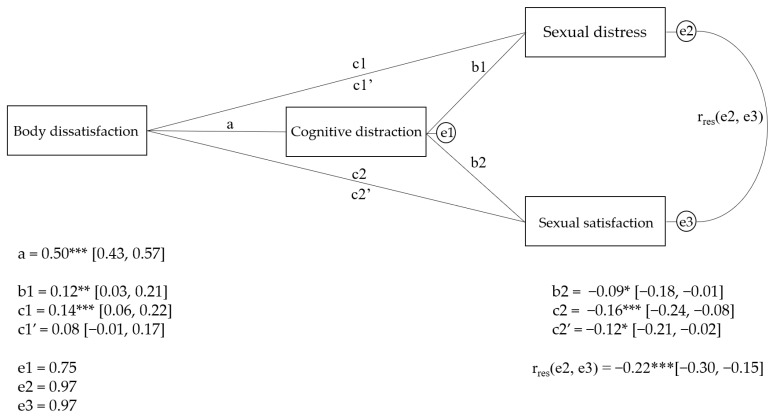
Standardised regression coefficients for the association between body dissatisfaction, sexual satisfaction, and sexual distress via cognitive distraction. The residuals of the model are referred to as the e’s. r_res_ represent the correlation between residuals. Values in brackets refer to the lower and upper limits of 95% CI. *** *p* < 0.001, ** *p* < 0.01, * *p* < 0.05.

According to Kline’s [57] criteria, only body appearance cognitive distraction showed a non-normal univariate distribution (Kurtosis > 10), which can violate the normality assumption required in mediation analysis. Therefore, we used the bootstrap approach as a robust method to address non-normality. Bootstrapping does not rely on distributional assumptions, producing more reliable estimates of the indirect effect [52,58]. To assess whether cognitive distraction mediates the relationship between body dissatisfaction and sexual satisfaction and distress, a mediation analysis was conducted. The results indicate that body image dissatisfaction significantly predicts cognitive distraction, as evidenced by path a in Figure 2 (a = 0.50; 95% CI [0.43, 0.57]). A significant total effect was found for both associations: between body image dissatisfaction and sexual distress (c1 = 0.14; 95% CI [0.06, 0.22]), as well as between body image dissatisfaction and sexual satisfaction (c2 = −0.16; 95% CI [−0.24, −0.08]). Concerning the completely standardised indirect effect of cognitive distraction as a mediator in the aforementioned associations, the effect was significant but reduced in both cases (ab1 for sexual distress = 0.06; 95% bootstrap CI [0.01, 0.13]; ab2 for sexual satisfaction = −0.05; 95% bootstrap CI [−0.10, −0.01]; 95%). These effects were small, suggesting that cognitive distraction plays a mediating role in the relationship between body image dissatisfaction and both sexual satisfaction and distress. Only 28% of the total effect of body image cognitive distraction on sexual satisfaction is accounted for by cognitive distraction (mediated proportion (MP) = 0.31). However, 43% of the total effect of body image cognitive distraction on sexual distress is mediated by cognitive distraction (MP = 0.43). According to Kenny et al. [59], this indicates partial mediation by cognitive distraction in the relationship between body image cognitive distraction and both sexual satisfaction and distress.

### 3.4. Moderated Mediation Model

To assess the role of age as a moderator in the mediation effect of cognitive distraction on the relationship between body image dissatisfaction and sexual satisfaction and distress, a moderated mediation analysis was conducted for each outcome variable. The results indicate that body image dissatisfaction was significantly associated with cognitive distraction; however, it was not significantly associated with either sexual satisfaction or sexual distress. This finding remained consistent when age was included as a moderator for both variables, as detailed in Table 4.

Body image dissatisfaction negatively predicts sexual satisfaction, and this relationship persists when age is included as a moderator. A similar pattern is observed for cognitive distraction as a predictor of sexual satisfaction. However, when age is included as a moderator, it does not have a predictive effect, as shown in Table 4.

Concerning the conditional indirect effect of body image dissatisfaction on sexual satisfaction, we observed a statistically significant moderation effect, wherein the cognitive distraction effect (indirect effect) between body image dissatisfaction and sexual satisfaction diminished as age increased. Specifically, for older men (+1 SD), the mediator effect of cognitive distraction became non-significant, indicating a potential protective role of age (see Table 5). Results for the conditional indirect effect on sexual distress revealed a significant indirect effect only for individuals of intermediate age (M = 35.32 years), with no evidence of mediation for younger men (−1 SD) or older men (+1 SD), as detailed in Table 5.

## 4. Discussion

The present study investigated both a mediation and a moderated mediation model to explore the relationship between body image dissatisfaction and sexual satisfaction and distress. Specifically, it examined body image cognitive distraction during sexual activity as a mediator variable, with age serving as a moderator variable. The analysis was conducted using a sample of Portuguese men aged 18 years or older. The results regarding the scores of the measures indicate that this sample is highly satisfied with their body image, has low levels of body image cognitive distraction during sexual activity and sexual distress, and is highly satisfied sexually. Comparatively to other Portuguese studies that used the same measures, the total mean scores for the Body Appearance Cognitive Distraction Scale and Global Body Dissatisfaction Scale for this study are below the ones found by Silva et al. [32], Carvalheira et al. [34] and Manão and Pascoal [17], indicating a more satisfied and less distracted sample. Concerning the mean total scores found for the Global Measure of Sexual Satisfaction, these are above the ones found by Pascoal et al. [54] for men in the non-clinic sample (M = 25.64, SD = 7.57), a result that once more corroborates that our sample is satisfied and has high levels of sexual health. As the measure of distress used in this study is an adaptation of the Natsal study conducted by Mercer et al. [56] and similar procedures have been used with samples of women [30], we cannot compare the results of sexual distress found in the current study.

The bivariate analysis of the associations between variables shows that they are associated with one another. The non-significant correlations observed between body image dissatisfaction and age suggest that body image dissatisfaction is independent of age, implying that men’s concerns about their body image are not influenced by their age. This finding may be attributed to the mean age of our sample, which could reflect a cohort that was socialised in an era less influenced by the current societal pressures related to beauty, particularly the emphasis on muscularity. Furthermore, the strong association observed between body image dissatisfaction and cognitive distraction during sexual activity is consistent with previous studies conducted on the Portuguese population, both with and without sexual problems (e.g., [32,34,60,61]). Our results reinforce what cognitive-based models suggest: the more focused men are on their body image, the bigger their cognitive distraction during sexual activity will be, and they will not be able to process sexual clues. Regarding the association between sexual distress and satisfaction, this relation is weak, with almost medium strength. This association is comprehensible, since the sexual satisfaction measure we have used evaluates the totality of sex life and the sexual distress measure is circumscribed to sexual distress with sexual function. There is some overlap, but not a total overlap, between the constructs since they may be influenced by varied factors, as suggested by Stephenson and Meston [19]. The present mediation analysis suggests that body image dissatisfaction is associated with body image cognitive distraction during sexual activity, which is consistent with previous research, including studies conducted with Portuguese samples [17,32,34,60], which identified body image dissatisfaction as a significant predictor of cognitive distraction related to body image during sexual activity. These findings are consistent with Nobre’s cognitive model of sexual response [62] and Barlow’s 1986 [29] cognitive–affective model for sexual dysfunction as well as with its updated version [29,63]. Concerning the significant total effect observed between body image dissatisfaction, sexual satisfaction, and distress, this finding aligns with previous research on the relationship between body image dissatisfaction and sexual satisfaction in women (e.g., 24), thereby reinforcing the notion that the effects observed in women are also applicable to men. Specifically, body image dissatisfaction affects sexual experiences in both men and women.

A negative association was identified between body image dissatisfaction and sexual satisfaction, which aligns with the existing literature indicating that body dissatisfaction negatively impacts individuals’ sexual satisfaction, thereby hindering optimal sexual health outcomes. In this regard, it can be hypothesised that body dissatisfaction in men impedes both physical and psychological well-being, affecting overall quality of life [19,21]. Furthermore, the positive association between body image dissatisfaction and sexual distress suggests that body dissatisfaction may serve as a significant clinical indicator, which must be notably present for the diagnosis of sexual dysfunction [19]. This highlights the potential need to consider body image dissatisfaction when addressing or investigating men’s sexuality. Body image-related cognitive distraction during sexual activity mediates the relationship between body image dissatisfaction and both sexual satisfaction and distress, thereby supporting the notion that attention to non-erotic cues influences men’s sexual experiences, as proposed in Barlow’s cognitive–affective model of sexual dysfunction [63].

Regarding the moderating role of age, which represents the most innovative contribution of the present study, we observed that age moderates the indirect effect of cognitive distraction on the relationship between body image dissatisfaction and sexual satisfaction. Specifically, older men exhibited a reduced effect of cognitive distraction on this relationship, suggesting a potential protective role of age. This finding aligns with the assertion of Tiggemann and McCourt [2], who posited that ageing men may develop mechanisms that allow them to shift their focus away from concerns about body image and instead become more appreciative of their health and functional capabilities.

Regarding the relationship between body image dissatisfaction and sexual distress, the mediation effect of cognitive distraction is moderated by age, but only in individuals of intermediate age (M = 35.52 years). This finding can be explained by the specific life stage of adulthood, where individuals may be more susceptible to societal pressures to conform to dominant masculine sexual scripts—portraying men as assertive and sex-driven—which may lead to performance-related concerns, particularly emphasising erectile functioning [12]. Consequently, focusing on sexual performance, these individuals are more vulnerable to experiencing anxiety-inducing thoughts during sexual activity [60,64], which may, in turn, exacerbate sexual distress. This pattern may not apply to older adults, as Santos-Iglesias et al. [36] suggest that, despite the potential for sexual difficulties, older adults rarely experience significant sexual distress.

## 5. Conclusions

In line with other studies, our study found that body image dissatisfaction, cognitive distraction, sexual distress, and sexual satisfaction are significatively associated with each other, contributing to a comprehensive view of sexual outcomes that considers both positive and negative results. Our results reinforce the idea that these constructs do influence each other and that body dissatisfaction may be a relevant clinical indicator when assessing and intervening with sexual dysfunction when considering its crucial multidimensional component, sexual distress [27]. It innovates by establishing that, in cis men, age has a differential effect on these associations, and as age increases, the effect diminishes; therefore, it suggests that age may be a protective factor for the associations found.

## 6. Study Limitations

There are several limits to this study. Firstly, since we used a non-probabilistic sample and the sociodemographic characteristics of the sample do not represent the majority of the male Portuguese population (e.g., the education level and socioeconomic status), the results cannot be generalised and need to be interpreted as such. Furthermore, the fact that the sample is highly satisfied and presents high levels of sexual satisfaction and low levels of sexual distress also limits the extent of our interpretation of the results.

Because we used self-report measures, which have limitations such as social desirability bias and the inability to capture the complexity of some constructs, to assess constructs related to human sexuality, which are known to be sensitive to this desirability, our results need to be interpreted with caution.

Finally, even though the results concerning age are a new contribution to the field, a larger sample that includes older men would improve our understanding of the model.

## 7. Future Directions

We suggest, as possible directions for future studies, the use of a more diverse sample, namely the inclusion of participants with other sexual orientations, to understand if the results that we found will also be found in the new sample, as more recent studies suggest that there are differences on how body image is experienced among heterosexual and homosexual men (see [12]). We also recommend that future studies include a clinical sample of men with sexual dysfunctions to test if the model is replicated with a good fit. While the current study examined age as a continuous moderator to capture its effect on the mediation process, future research could explore model invariance across distinct age groups. Conducting multigroup analyses would allow for a deeper understanding of whether the mediation process differs qualitatively across age categories. This approach could provide a better comprehension of how age shapes the relationships examined in this model and whether the underlying mechanisms vary significantly across developmental or life-stage groups. A longitudinal design would allow us to see how the association of variables evolve and to establish causality, as in the current study the causal model is theoretically based but not demonstrated due to the cross-sectional nature of the design. Finally, we consider that it is necessary to explore if the effect found is due to specific processes related to ageing or related to the role sexual function plays in one’s life and how these may interact with media and societal portraits of sexuality of body image across ageing. We consider that a qualitative study that explores the relationship between body image and sexuality among ageing people may shed light on these processes.

## 8. Clinical Implications

Our study suggests that body image concerns during sexual activity should be considered in different clinical contexts where men are assessed and intervened in all age frames, since age seems to influence body image dissatisfaction and, subsequently, sexual satisfaction and distress.

## Figures and Tables

**Table 1:** Sociodemographic characteristics of the sample.

	Total Sample (N = 597)
	*n* (%)
Relationship status	
Married	213 (35.70)
Living in common-law relationship/cohabiting	131 (21.90)
Dating	231 (38.70)
Separated	6 (1)
Divorced	16 (2.70)
Education	
6th grade	2 (0.30)
9th grade	25 (4.20)
10th–12th	70 (11.70)
University frequency	114 (19.10)
Undergraduate/master’s degree	356 (59.60)
PhD	30 (5.00)
Ethnicity	
Caucasian	588 (98.50)
Black	4 (0.70)
Other	5 (0.80)
Socioeconomic status	
High	386 (64.70)
Medium	198 (33.20)
Low	10 (1.70)
Do not know	3 (0.50)
Area of residency	
North	92 (15.40)
Centre	93 (15.60)
Greater Lisbon Area	344 (57.60)
Algarve	19 (3.20)
Alentejo	16 (2.70)
Madeira Island	9 (1.50)
Azores Island	1 (0.20)
Overseas	23 (3.90)
	M (SD)
Age	35.52 (8.78)

**Table 2:** Measures of central tendency, dispersion, and distribution for the scales used in this study.

Scale	M	SD	Min	Max	Skewness	Kurtosis
Global Body Dissatisfaction	4.71	3.21	0	20	1.08	2.54
Body Appearance Cognitive Distraction	12.62	3.95	10	47	3.36	17.72
Sexual Distress	1.92	2.97	0	18	2.14	5.96
Global Measure of Sexual Satisfaction	28.64	6.00	10	35	−1.00	0.34

**Table 3:** Pearson’s bivariate correlation between study variables.

	1	2	3	4	5
Body dissatisfaction	-	0.50 ***	−0.16 ***	0.14 ***	−0.08
2. Cognitive distraction	-	-	−0.15 ***	0.16 ***	−0.10 ***
3. Sexual satisfaction	-	-	-	−0.25 ***	−0.17 ***
4. Sexual distress	-	-	-	-	0.06
5. Age	-	-	-	-	-

Note: *** *p* < 0.001.

**Table 4:** Results of moderated mediation analyses.

	Mediation Model (Cognitive Distraction)		Dependent Model I (Sexual Satisfaction)		Dependent Model II (Sexual Distress)	
	*Beta*	SE	*Beta*	SE	*Beta*	SE
Constant	−0.00 [−0.07, 0.07]	0.04	−0.00 [−0.08, 0.07]	0.04	0.00 [−0.08, 0.08]	1.63
Cognitive distraction	-	-	−0.11 [−0.20, −0.02]	0.05	0.13 [−0.04, 0.22]	0.05
Body image dissatisfaction	0.50 [0.43, 0.57]	0.04	−0.11 [−0.20, −0.02]	0.05	0.07 [−0.02, 0.17]	0.05
Age	−0.06 [−0.13, 0.01]	0.04	−0.20 [−0.28, −0.12]	0.04	0.09 [0.01, 0.17]	0.04
Body image dissatisfaction × Age	−0.03 [−0.09, 0.03]	0.03	−0.10 [−0.19, −0.03]	0.04	0.09 [0.01, 0.17]	0.04
Cognitive distraction × Age	-	-	0.04 [−0.05, 0.14]	0.05	−0.03 [−0.12, 0.07]	0.05
*R* ^2^	0.25		0.08		0.05	

Note: Values in brackets refer to lower and upper limits of 95% CI. SE = standard error.

**Table 5:** Conditional indirect effects of cognitive distraction on sexual satisfaction and sexual distress across age groups.

Moderator (Value)	Conditional Indirect Effect	Bootstrap SE	Bootstrap LLCI	Bootstrap ULCI
Dependent variable = sexual satisfaction; Mediator = cognitive distraction				
−1 (26.74 years)	−0.08	0.03	−0.15	−0.02
M (35.52 years)	−0.05	0.02	−0.10	−0.01
+1 (44.29 years)	−0.03	0.03	−0.11	0.03
Dependent variable = sexual distress; Mediator = cognitive distraction				
−1 (26.74 years)	0.08	0.05	−0.03	0.08
M (35.52 years)	0.07	0.01	0.01	0.15
+1 (44.29 years)	0.05	0.06	−0.05	0.19

Note: The resampling method of bootstrap was with 10,000 samples using a 95% CI. SE = standard error; LLCI = lower level of confidence interval; ULCI = upper level of confidence interval.

## Data Availability

The datasets presented in this article are not readily available because of privacy considerations. The raw data supporting the conclusions of this article will be made available by the authors upon reasonable request.
